# Rethinking the Psychogenic Model of Complex Regional Pain Syndrome: Somatoform Disorders and Complex Regional Pain Syndrome

**DOI:** 10.5812/aapm.7282

**Published:** 2012-09-13

**Authors:** Renee J. Hill, Pradeep Chopra, Toni Richardi

**Affiliations:** 1 Center for Psychological Studies, Nova Southeastern University, Davie, Florida, USA; 2 Brown Medical School, Brown University, Rhode Island, Connecticut, USA; 3 Center for Psychological Studies, Nova Southeastern University, Fort Lauderdale, Florida, USA

**Keywords:** Complex Regional Pain Syndromes, Somatoform Disorders, Conversion Disorder, Pain Disorders, Depression, Anxiety, Cognitive Therapy

## Abstract

**Abstract:**

Explaining the etiology of Complex Regional Pain Syndrome (CRPS) from the psychogenic model is exceedingly unsophisticated, because neurocognitive deficits, neuroanatomical abnormalities, and distortions in cognitive mapping are features of CRPS pathology. More importantly, many people who have developed CRPS have no history of mental illness. The psychogenic model offers comfort to physicians and mental health practitioners (MHPs) who have difficulty understanding pain maintained by newly uncovered neuro inflammatory processes. With increased education about CRPS through a biopsychosocial perspective, both physicians and MHPs can better diagnose, treat, and manage CRPS symptomatology.

## 1. Introduction

Complex Regional Pain Syndrome (CRPS) is a neuropathic disorder that usually occurs after a trauma, surgery, medical procedure, prolonged immobilization (1). CRPS I is characterized by intractable pain that is out of proportion to the trauma (e.g., sprains, electrical burns, hairline fractures, and immobilization), and CRPS II is characterized by unrelenting pain that occurs subsequent to a nerve injury (1). The criteria for diagnosing CRPS is abstruse due to the vast spectrum of disease presentation and can include, but is not limited to: intractable pain out of proportion to an injury; intense burning pain; pain from non-injurious stimulation; an exaggerated feeling of pain; temperature changes in the affected body part; edema; motor/trophic disturbances; changes in skin, hair, and nails; and abnormal skin color (2). These symptoms often begin in a limb of CRPS I patients and spread bilaterally or systematically to adjacent limbs (1). Many healthcare providers are not familiar with the presentation of CRPS and, although the disease may be under reported, more than 50,000 new cases of CRPS I occur annually in the United States (3). We suspect that a significantly large number of cases of CRPS are being missed by their health care providers, most of them being attributed to psychogenic issues rather than pain from CRPS. Receiving a CRPS diagnosis in the absence of visible signs of the disease is rare and inexperienced physicians will often refer these patients for psychological evaluations without providing pain management. In addition, when CRPS patients do not heal normally or respond to medical treatments as expected, physicians often label their symptoms as “psychogenic pain” (1) rather than acknowledge their training, educational, and clinical limitations. Physicians may deem CRPS symptoms as emotionally based (i.e., psychogenic pain), due to the puzzling nature of the syndrome presentation (4), which can fluctuate erratically. Physicians’ psychogenic conceptualization of CRPS symptoms may be considered invasive and unhelpful to the CRPS patient (5) in need of pain management and rehabilitation. Given the limitations of medical interventions to effectively treat the source of many pain complaints and the practice of physicians referring “difficult” patients for psychological evaluations without pain treatment, MPHs should be identify physical pain from psychogenic pain and be familiar with psychological symptoms associated with chronic pain diseases such as CRPS. Clarification regarding whether or not psychological problems precipitate or influence the onset of CRPS may aid MHPs in gaining an accurate mental status of the patient and can help MHPs provide appropriate care. In addition, educating MHPs about CRPS pathology may prevent clinicians from misdiagnosing patients with an erroneous disorder (e.g., conversion disorder), which can ultimately prevent patient’s from receiving treatments found to prevent disease progression. Intellectual, perceptual, and psychological disturbances that are associated with CRPS pathology should be explained to MPHs, so they can provide an accurate diagnosis and treatment plan for CRPS patients.

## 2. Rethinking the Psychogenic Model of CRPS

As previously mentioned, psychological factors have been suspected in solely initiating or causing CRPS symptoms (1). Suspecting that psychological factors are entirely responsible for influencing CRPS carries the implication that pain is all in the patient’s head, and ultimately blames the patient for their pain. This inaccurate assumption is damaging to people with CRPS who are in desperate need for appropriate care and treatment interventions by their physicians. As aforementioned, patients with symptoms of chronic pain that are resistant to treatment and in excess of what physicians would expect for the presenting injury are often referred for a psychological evaluation. Mental health professionals are educated about Somatoform disorders, but lack knowledge about neurological disorders such as CRPS and fibromyalgia. CRPS should always be considered as an alternate diagnosis to a somatoform Disorder, if the patient presents with pain elicited by an injury that appears out of proportion to the trauma or symptoms of pain that cannot be explained by standard models of pain etiology. A Somatoform disorder is characterized by physical symptoms that mimic physical disease or injury for which there is no identifiable organic cause, or if there is an identifiable cause for physical symptoms, such as pain or neurological deficits, the symptoms cannot be fully explained by the nature of the injury and are thought to be psychologically maintained. Symptoms resulting from a Somatoform disorder are said to be due to the manifestation of mental distress or have a psychogenic origin (6). Symptoms of body and sensory disturbances common in CRPS pathology are often mistaken for somatoform disorders, particularly conversion disorder or pain disorder. The main CRPS criterion is that the pain must be out of proportion to the trauma and this unfortunately is also the same criterion for pain disorder; a type of somatoform disorder. A workforce has been established for the new edition of the DSM to redefine the nomenclature of somatoform disorders to reduce incidences of misdiagnosis of medical conditions (e.g., CRPS, fibromyalgia, and lupus) with somatoform disorders. It is sought to investigate if psychological factors are correlated with the onset of CRPS (7). They conducted a qualitative systematic review utilizing 31 empirical studies exploring psychological factors and CRPS. Due to the studies’ poor to moderate methodological quality, the researchers interpreted the data with caution. They found prospective studies revealed no relationship between depressions, anxiety, or neuroticism and CRPS I. Retrospective and cross-sectional studies reviewed were inconclusive due to the contradictory findings. Although some studies did not support the psychogenic model, researchers discovered a higher pain day (i.e., CRPS flare-up) was predictive of higher depression, anxiety, and anger scores. Conversely, (8) reported that depression and anxiety were predictors of greater pain levels for their subjects. Harden et al. investigated CRPS symptoms following total knee arthroplasty (9). The authors found greater pre-operative anxiety is significantly associated with a CRPS diagnosis at a 1-month follow-up. However, the authors did not point out that people with greater pre-operative anxiety may be experiencing more severe pain levels and conceivably have dysautonomias, which are both features of CRPS. Pre-operative anxiety was not found to be a significant predictor of a CRPS diagnosis at a three and six month follow-up; thus elucidating that there is a relationship between anxiety and CRPS, although it appears to be non-causal. In contrast to the psychogenic model, (9) found that pre-operative depression was not a significant predictor of CRPS. Furthermore, the researchers found baseline pain intensity did not predict an initial post-surgery diagnosis, but was a predictor of a CRPS diagnosis at a three and six month follow-up. Additionally it is discovered that participants with greater CRPS severity at a six month and 12-month follow-up had post-surgical increases in depression (10). This showcases that depression is most likely a consequence of chronic pain rather than a causal agent in CRPS pathology. Intuitively, they also found that pain intensity was positively associated with greater CRPS severity at a 6-month follow-up. It is confirmed that the way in which psychological factors interact with CRPS is highly complex (8). However, they found that CRPS patients are often trapped in a vicious cycle where pain creates emotional distress that provokes disuse of the affected limb(s) through vegetative processes, which in turn causes more pain. This cycle may be a maintaining factor of the disease, and provides support for associations between pain intensity and concurrent psychological distress in the (9, 10) studies. Also it is systematically reviewed five studies assessing the relationship between insomnia and CRPS I (7). In two studies, no differences in insomnia were found between the CRPS I group and the control group; however, two studies indicated a higher incidence of insomnia in CRPS I patients versus the control group. More studies are needed to assess for a relationship between CRPS I and insomnia in order to establish a stronger relationship between neuropathic pain and sleep disturbances. However, it is logical that pain and related discomfort would interfere with sleep onset and sleep maintenance; therefore, replication of these studies may indicate a directional correlation between CRPS and insomnia. Based on limited research in the area, it appears that psychological factors play a role in CRPS, but the exact relationship is unknown. Several studies have indicated pain predicts higher depression, anxiety, and anger; and (8) postulate that pain influences psychological symptoms, which in turn exacerbates pain symptoms. There is no indication that psychological factors cause the onset of pain in CRPS patients. Given these findings, it appears appropriate for MHPs to assess patients who meet the criteria set forth by the International Association for the Study of Pain’s (IASP) (see Appendix A; (11)) CRPS diagnosis for mood and anxiety disorders. If the patient has a comorbid mood or anxiety disorder, pharmacological treatment should not be restricted to palliative care focusing on pain management (e.g., analgesics). Pharmacotherapy should also treat the patient’s depression and anxiety (e.g., SNRIs or tricyclics); as depression and anxiety can exacerbate pain (8). With the presence of a comorbid sleep disorder, or any other concurrent psychological disorder, pharmacotherapeutic interventions should be implemented that avoid prescribing the patient more medications than clinically necessary (i.e., poly-pharmacy). For example, according to (12), choosing a sedating antidepressant or an atypical antidepressant may alleviate neuropathic pain, reduce depression and anxiety symptoms, and may induce sleep onset. This will reduce interactions and long-term side effects from poly-pharmacy. The literature review revealed psychological interventions, that increase parasympathetic nervous system activation such as autogenic training, hypnotherapy, guided imagery, progressive muscle relaxation, and thermal biofeedback may help reduce anxiety and depression as well as allay CRPS related symptoms (i.e., pain, temperature, blood flow) (8). The direction of the treatment effects are unclear, as research has not elucidated whether therapies ameliorate pain by reducing anxiety and depression or if these interventions reduce psychological symptoms that maintain pain and pain related behaviours. Nevertheless, reducing activation of the sympathetic nervous system through implementing relaxation techniques seems promising considering the large dysautonomic component in CRPS pathology. Research supports that CRPS symptomatology is best managed when psychological and pharmacotherapeutic interventions are introduced concurrently with medical interventions such as pain management and physical and occupational therapies.

## 3. Neurological Deficits Associated with CRPS

The psychogenic model cannot account for the neurological and neuroanatomical deficits related to CRPS pathology. Schwartzman et al. investigated these purported deficits in CRPS patients with an extensive neuropsychological battery (13). Approximately 64.95% of the CRPS patients had a compromised ability to perform higher order mental manipulations. 42.33% of these compromised patients produced mildly reduced to low average scores an all executive tests, and 22.62% produced mildly reduced scores on all executive tests in addition to performing poorly or in the borderline range of declarative memory tests measures. Concurrent use of FMRIs and PET scans could have indicated whether neuroamatomical loss was associated with the patients’ compromised higher order mental manipulation abilities. Gehat et al. performed CRPS post-mortem research that indicated gross abnormalities and atrophy of the gray and white matter in regions of the brain involved in pain perception, emotional experience, and autonomic functions as compared to controls (14). The neuroanatomical loss associated with CRPS pathology, may explain the difficulties CRPS patients had during assessments of working memory and executive functioning in the (13) study. The psychogenic model cannot account for these aforementioned brain abnormalities. Research done by (15) indicated chronic pain patients, including those with CRPS, have difficulty making emotional decisions. In a gambling task, CRPS patients showed no improvement over time, unlike other chronic pain patients. Interestingly, in CRPS patients, their cognitive abilities seemed independent of their pain, providing support for (14) that brain abnormalities and neurological deficits may be found in CRPS patients. The (15) study showed other cognitive abilities such as short-term memory, attention, and general intelligence were within normal limits of the general chronic pain population. Thus, differences in cognitive abilities appear to be present between CRPS patients and the chronic pain population. Investigating whether test performance improves as a function of decreased pain levels could also help clarify the cause of CRPS patients’ compromised ability to perform higher order mental manipulations. Reasonably, the ability to attend to stimuli and consolidate information could be compromised by medications used in conjunction with pain that may cause retrograde amnesia (e.g., Tramadol, benzodiazepines, anticonvulsants, analgesics). Developing cognitive treatments that prevent or reduce deficits in neuropsychological functioning are indicated. Moreover, it would be interesting to assess whether increasing cognitive performance on higher order mental manipulations (e.g., teaching chunking techniques) reduces depression and pain ratings.

## 4. Disturbances in Cognitive Mapping and Body Perception

Lewis et al. investigated if disturbances in body perception contributed to pain and disabilities in people with CRPS (16). Repeated themes about body perception were identified among CRPS patients. They consisted of the following: hostile feelings towards the affected limb (“I’m disgusted my arm is this way”), disparity between what is apparent and what is felt in the affected limb (“I actually feel as if my finger tips are my knuckles”), distorted mental image the affected limb (“I can see my big toe and I can’t see anything else from the knee down”), spectrum of dissociation about the affected limb (“It was just like this foreign body you were carrying around with you”); and conscious attention of the affected limb (“I used to try to hide it”). These thoughts regarding body dissociation cause patients to fail to pay attention or care for their affected limb and they are often recognized as neglect-like symptoms (17). Frettloh et al. also had similar findings, as they found that CRPS patients were significantly more likely than other chronic pain patients to exhibit neglect-like symptoms (17). Moreover, CRPS patients were more likely than other chronic pain patients to describe their limb in a de-personalized manner. In continuation of elucidating body part distortions, (18) studied CRPS patients whose hands were affected by the disease. 54.4% of the CRPS patients reported their hand was “foreign” or “strange” (e.g., “This is not my hand,” “This hand feels like the hand of another person”). 48 percent of the CRPS patients also had an impaired ability to identify fingers on the affected hand compared to the non-affected hand. These findings leave a lot of questions to be answered; especially to those that are speculative about the legitimacy of CRPS. It is plausible that deficits in cognitive mapping account for these bodily distortions. However for those that supports the psychogenic model of CRPS, these disturbances in body perception could be related to a type of body dysmorphic disorder. If these behavioural manifestations were related to dysfunctions in self-image, than mirror therapy, which is often used successfully to correct the neurofeedback of the affected part would not likely be efficacious. Nevertheless, it is a priority to discern how these disturbances contribute to pain perception and how these distortions can be treated. Further research investigating the etiology and prevalence of these cognitive distortions is warranted. Immobilization of a limb (which is one of the causes of CRPS; (1)) can create changes in functioning and perception of the limb. When an injury elicits a pain response, guarding the affected site is an innate response to prevent further injury; If the pain is unrelenting, than this guarcling behavior immobilizes the limp. It is plausible that immobilizations of a healthy limb, either due to medical interventions or protective mechanisms, would eventually cause disturbances in body perception through neural feedback mechanisms. Feedback from the limb’s disuse may initiate changes in neural pathways which are thought to initiate trophic changes, temperature changes, and aggressive immune responses that ultimately contribute to increased pain perception (19). Additionally, disuse of the affected limb due to pain related immobilization can lead to a fear of movement, which in turn begins the process of pain sensitization (20). Cryotherapy, which is used to combat post-surgical inflammation, is also hypothesized to also interfere with sensory and motor neural pathways and to create disturbances in body perception (1). Investigating if immobilization of healthy limb causes CRPS type pathology is unethical to perform on humans, so more creative research designs are needed. Wen-Wu et al. studied a rat whose leg had been immobilized after a tibia fracture (21). The rat developed biological changes similar to those experienced by patients with CRPS (e.g., elevated enzyme levels, vascular changes, and bone changes). Replications of this study and other creative research designs would be beneficial to help reduce the number of CRPS cases caused by immobilization of a limb. Treatments that correct feedback dysfunctions, such as those seen with the immobilization of a limb, through the use of visual input (e.g., mirror therapy) are hypothesized to reduce pain, cognitive distortions surrounding pain, and dysfunction (e.g., disuse and disabilities). In (19) study, mirror therapy was shown to reduce pain stiffness and dysfunction in the early stages of CRPS I. However, these effects were minimal with chronic or trophic presentations of the disease.

## 5. Discussion

There is compelling evidence against the psychogenic model of CRPS onset. Researchers have been exploring the hypothesized causes and subsequent disorder surrounding this elusive syndrome. Based on limited research in the area, it appears that psychological factors play a role in CRPS, but the exact relationship is unknown. Several studies have indicated that chronic pain predicts higher depression, anxiety, and anger; and (8) postulate that pain influences psychological symptoms which in turn exacerbates pain symptoms. However, there is no indication that psychological factors cause the onset of pain, autonomic dysfunction, and movement disorders in CRPS patients. Given these findings, it appears appropriate for Mental Health Professionals to assess patients who meet the CRPS criteria set forth by International Association for the Study of Pain (IASP) for mood and anxiety disorders. An assessment to rule out somatoform disorders is also needed, as CRPS symptoms have overlapping diagnostic criteria. Most importantly, prompt treatment of CRPS is important for good prognostic outcomes, yet in most centers, there can be unacceptably long waits for appointments at specialty pain clinics, or with other specialists. In most cases there is a significant delay in starting appropriate and aggressive treatment due to a lack of experience on the part of the physicians in recognising CRPS. Ideally, mechanisms should be in place to ensure CRPS patients with prompt assessment by specialist physicians. Given the increasing number of CRPS cases (3) all health care practitioners and MHPs should attend educational seminars about CRPS to understand CRPS onset is not likely due to psychogenic factors. These seminars should provide an overview of: (a) the hypothesized causes of CRPS; (b) the different symptom presentations; (c) appropriate diagnostic assessments (e.g., Beck Depression Inventory, Beck Anxiety Inventory; Wechsler Adult Intelligence Scale, McGill Pain Questionnaire); medical referrals (e.g., neurologist or pain clinic); and (e) psychological treatment interventions (e.g., autogenic training, progressive muscle relaxation, hypnotherapy, and biofeedback). There is also a need for assessing psychotherapy’s efficacy in helping people with CRPS manage intense pain and disability.
